# Facile Synthesis of Oxygen-Doped g-C_3_N_4_ Mesoporous Nanosheets for Significant Enhancement of Photocatalytic Hydrogen Evolution Performance

**DOI:** 10.3390/ma17133345

**Published:** 2024-07-06

**Authors:** Tiekun Jia, Jingjing Li, Zhao Deng, Dongsheng Yu, Joong Hee Lee

**Affiliations:** 1 School of Materials Science and Engineering & Henan Province International Joint Laboratory of Materials for Solar Energy Conversion and Lithium Sodium Based Battery, Luoyang Institute of Science and Technology, Luoyang 471023, China; yudongsheng@lit.edu.cn; 2 State Key Lab of Materials Synthesis and Processing, Wuhan University of Technology, Wuhan 430070, China; dengzhao@whut.edu.cn; 3 Department of Nano Convergence Engineering, Jeonbuk National University, Jeonju 54896, Republic of Korea; jhl@jbnu.ac.kr

**Keywords:** O doped, gC_3_N_4_, nanosheets, porous structure, hydrogen evolution

## Abstract

In this work, oxygen-doped g-C_3_N_4_ mesoporous nanosheets (O-CNS) were synthesized via a facile recrystallization method with the assistance of H_2_O_2_. The crystal phase, chemical composition, morphological structure, optical property, electronic structure and electrochemical property of the prepared O-CNS samples were well investigated. The morphological observation combined with the nitrogen adsorption–desorption results demonstrated that the prepared O-CNS samples possessed nanosheet-like morphology with a porous structure. Doping O into g-C_3_N_4_ resulted in the augmentation of the specific surface area, which could provide more active sites for photocatalytic reactions. Simultaneously, the visible light absorption capacity of O-CNS samples was boosted owing to the regulation of O doping. The built energy level induced by the O doping could accelerate the migration rate of photoinduced carriers, and the porous structure was most likely to speed up the release of hydrogen during the photocatalytic hydrogen process. Resultantly, the photocatalytic hydrogen production rate of the optimized oxygen-doped g-C_3_N_4_ nanosheets reached up to 2012.9 μmol·h^−1^·g^−1^, which was 13.4 times higher than that of bulk g-C_3_N_4_. Thus, the significantly improved photocatalytic behavior was imputed to the synergistic effect of the porous structure, the increase in active sites, and the enhancement of visible light absorption and charge separation efficiency. Our research highlights that the synergistic effect caused by element doping will make a great contribution to the remarkable improvement in photocatalytic activity, providing a new inspiration for the construction of novel catalysts.

## 1. Introduction

Photocatalytic hydrogen production is an advanced technology that can convert renewable solar energy into clean hydrogen energy. In terms of photocatalysis, the photocatalyst is a pivotal factor affecting the efficiency of photocatalytic hydrogen evolution. Thus, it is crucial to develop an efficient, stable and low-cost photocatalyst for facilitating the rapid development of photocatalytic hydrogen evolution [1]. Graphitic carbon nitride (g-C_3_N_4_) is a new type of organic semiconductor with a bandgap of about 2.7 eV, which is regarded as a promising photocatalytic material due to its nontoxicity, element abundance, suitable bandgap structure, stable physicochemical properties and good photoelectrochemical performance [2,3,4]. However, single-component g-C_3_N_4_ still has some inherent defects, such as a small specific surface area, limited solar light utilization and a long transfer distance of photogenerated charge [5,6], which seriously limit its potential application in photocatalytic hydrogen production.

By now, researchers have explored some effective modification methods to address the aforementioned shortcomings and improve the photocatalytic hydrogen production behavior of g-C_3_N_4_, such as element doping [7], morphology modulation [8], noble metal deposition [9] and forming composites with other materials [10,11,12]. Amongst these modification methods, element doping has been extensively accepted and adopted to improve catalytic activity due to its easy operation and simple control. As is well known, the atomic radius of the O element is analogous to that of C and N elements, and thus, it is an intriguing challenge to adopt oxygen doping for modifying g-C_3_N_4_ with enhanced photocatalytic hydrogen evolution performance. In previous studies, Shi’s group [13] conducted an investigation into the thermal gas shocking synthesis and photocatalytic performance of 2D ultrathin oxygen-doped g-C_3_N_4_ photocatalysts, and found that the electronic structure of oxygen-doped g-C_3_N_4_ photocatalysts was optimized and the carrier separation rate was significantly elevated. Jia and his coworkers [14] prepared S- and O-co-doped g-C_3_N_4_ nanosheets through a two-step annealing process with melamine as the g-C_3_N_4_ precursor for efficient hydrogen generation. Tang’s group [15] developed a defect-engineering approach to prepare O dopants and N defects in a g-C_3_N_4_ framework. Saka and his coworkers [16] prepared oxygen-doped g-C_3_N_4_ through a pyrolysis route combined with the treatment of HNO_3_ solution. All of these achievements in oxygen-doped g-C_3_N_4_ are deeply impressive; however, the processes mentioned above are most complicated and relatively highly energy consuming. Thus, it is especially desirable to explore an in situ and green strategy for achieving oxygen-doped g-C_3_N_4_ with improved photocatalytic hydrogen evolution behavior.

In this work, oxygen-doped g-C_3_N_4_ porous nanosheets with a large specific surface area were synthesized via a simple recrystallization method with H_2_O_2_ as the dopant source. The structure, morphology, chemical composition, optical absorption, specific surface area and carrier separation of the photocatalysts were comprehensively characterized. The photocatalytic activity and stability of the photocatalysts were evaluated in terms of hydrogen production under visible light illumination. In addition, the possible mechanism of the enhanced photocatalytic hydrogen evolution performance is proposed in this work. Up to now, there are limited reports on the investigation of photocatalytic hydrogen production in oxygen-doped g-C_3_N_4_ porous nanosheets.

## 2. Materials and Methods

### 2.1. Materials

All of the starting materials, including urea ((NH_2_)_2_CO), hydrogen peroxide solution (H_2_O_2_), chloroplatinic acid (H_2_PtCl_6_), triethanolamine (TEOA) and sodium sulfate (Na_2_SO_4_) were of analytical grade and directly used without further purification.

### 2.2. Preparation of g-C_3_N_4_ (u-CNB) and O-Doped g-C_3_N_4_ (O-CNS) Samples

#### 2.2.1. Preparation of g-C_3_N_4_ (u-CNB)

The bulk g-C_3_N_4_ photocatalyst was prepared by thermal condensation polymerization. A total of 10 g of urea was placed in the semi-closed corundum crucible and calcined in a muffle furnace at 550 °C for 2 h at a rate of 2 °C∙min^−1^. After being quickly cooled to ambient temperature, the obtained light-yellow product was taken out and fully ground into powder for further use.

#### 2.2.2. Preparation of O-Doped g-C_3_N_4_ (O-CNS)

In a typical process, 10 g of (NH_2_)_2_CO was completely dissolved in different volumes of commercial H_2_O_2_ (30 vol%) solution under ultrasound, and then dried at 80 °C for 15 h to recrystallize (NH_2_)_2_CO. Subsequently, the recrystallized (NH_2_)_2_CO was placed in a quartz tube and heated at 550 °C for 2 h at a rate of 2 °C∙min^−1^ under an Ar environment. Finally, the calcined products were ground and collected, and alternately washed with dilute nitric acid solution (1 mol·L^−1^), deionized water and ethanol to obtain O-CNS samples. The volumes of H_2_O_2_ solution were 57.5 mL, 60 mL, 62.5 mL, 65 mL, 67.5 mL and 70 mL, so the obtained O-CNS samples are, respectively, expressed as 57.5 O-CNS, 60 O-CNS, 62.5 O-CNS, 65 O-CNS, 67.5 O-CNS and 70 O-CNS.

### 2.3. Characterization

The X-ray diffraction (XRD) patterns of the as-prepared photocatalysts were obtained by using a Bruker D8 advance diffractometer (Bruker, Billerica, MA, USA) with Cu Ka radiation. X-ray photoelectron spectroscopy (XPS) was recorded on an ESCALAB 250 Xi spectrometer (Thermo Fisher Scientific, New York, NY, USA). The scanning electron microscopy (SEM) observation was performed on a Hitachi S-4800 electron microscope (Hitachi, Toyko, Japan) with an accelerating voltage of 5 kV. Transmission electron microscopy (TEM) and scanning transmission electron microscopy (STEM) were conducted to investigate the porous structure of the obtained O-CNS sample on a FEI Talos F200S transmission electron microscope (Thermo Fisher Scientific, New York, NY, USA) with a voltage of 200 kV. Brunauer–Emmett–Teller (BET) specific surface areas were recorded on a Tristar II 3020 surface area analyzer (Micromeritics, Norcross, GA, USA). UV–visible diffuse reflectance spectra (DRS) were collected on a TU-1901 UV–Vis spectrometer (Puxi, Beijing, China). Electron paramagnetic resonance (EPR) spectra were measured on a Bruker MEXnano spectrometer (Bruker, Karlsruhe, Germany).

### 2.4. Photoelectrochemical Measurements

The photoelectrochemical properties of the obtained samples were tested in a Na_2_SO_4_ electrolyte solution (0.5M, pH = 6.8) through a three-electrode system, which is equipped with a counter electrode (Pt sheet), a reference electrode (Ag/AgCl solution) and a working electrode (FTO conductive glass with spin-coated photocatalysts) [17]. A xenon lamp (300 W) equipped with a 420 nm cutoff quartz optical filter was taken as the visible light source in the photoelectrochemical measurements. The details for the measurements are already reported in our previous work [18].

### 2.5. Photocatalytic Hydrogen Evolution Experiments

Photocatalytic hydrogen production experiments were conducted in a closed-glass gas circulation system (Labsolar III AG), which was connected to an Agilent 7890B gas chromatograph (Agilent, Stevens Creek Blvd Santa Clara, CA, USA) for online testing. A PLS-SXE-300W xenon lamp (Perfectlight, Beijing, China) with a 420 nm cutoff filter was used as the visible light source. The vertical distance from the xenon lamp light to the surface of the mixed solution was 10 cm and the inner diameter of the reactor was 8 cm. A total of 50 mg of the photocatalyst was uniformly dispersed in the 100 mL aqueous solution containing 20% triethanolamine (TEOA) and H_2_PtCl_6_ solution (1 wt%), which were, respectively, used as sacrificial agent and co-catalyst. Every half hour, the xenon light source was turned on and the amount of hydrogen generation was detected each time.

## 3. Results

### 3.1. Structure and Morphology

**Figure 1:**
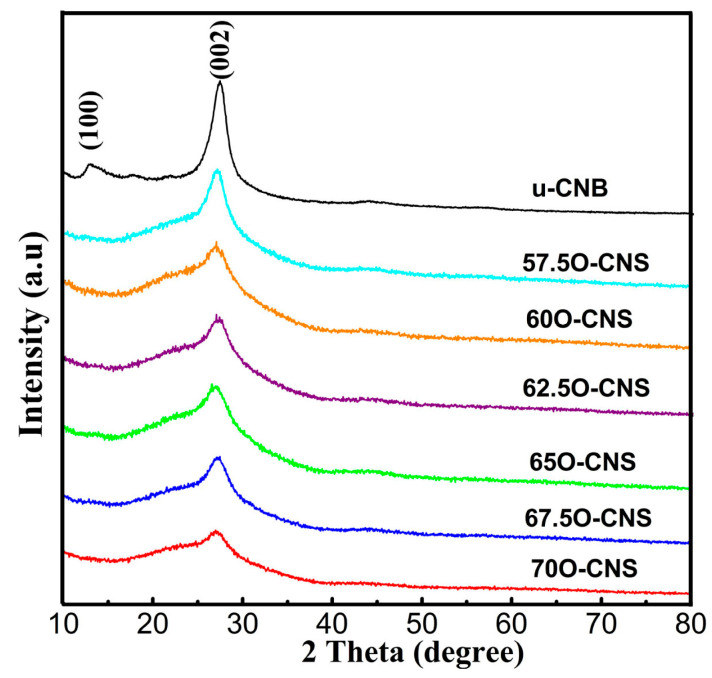
XRD patterns of uCNB and OCNS samples.

The effect of the amount of H_2_O_2_ on the crystal structure of the as-obtained samples was analyzed by XRD and the results are shown in Figure 1. It can be seen that the u-CNB sample exhibited two clear and sharp diffraction peaks near 13.2° and 27.8°, corresponding to the (100) and (002) crystal planes of the g-C_3_N_4_, respectively [19,20]. However, after incorporating O atoms into the g-C_3_N_4_ crystal, the diffraction peaks at 13.2° for all O-CNS samples disappeared. In addition to the above, the diffraction peak around 27.8° of the O-CNS samples became wider and weaker than that of u-CNB, and the intensity gradually decreased with the increase in H_2_O_2_ content, suggesting that the introduction of O atoms affected the structure of g-C_3_N_4_ crystals.

**Figure 2:**
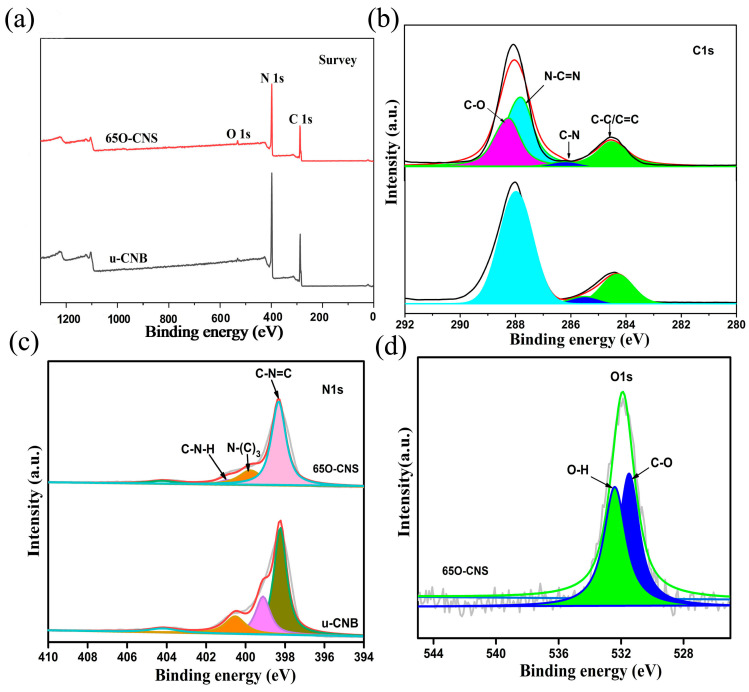
(**a**) Survey spectra, (**b**) C 1s spectra and (**c**) N 1s spectra of uCNB and 65 OCNS; (**d**) O 1s spectra of 65 OCNS.

As presented in Figure 2, the elemental composition and chemical status of the synthesized samples were characterized by XPS. From the survey spectra (Figure 2a), three strong signals for C, N and O elements appeared for the 65 O-CNS sample, indicating the existence of the O element. The weak signal of O 1s in u-CNB mainly originated from the adsorbed oxygen in the XPS measurement. Interestingly, the intensity of the C 1s and N 1s peaks in the 65 O-CNS sample was weaker than that in u-CNB, presumably due to the doping of the O element. Figure 2b–d show the high-resolution XPS spectra. Two dominant peaks for C 1s are situated around 284.6 and 288 eV (Figure 2b), which could be deconvoluted into four peaks centered at 288.2, 287.8, 286.2 and 284.5 eV, respectively, originating from C=O groups, N-C=N groups, C-N groups and C-C/C=C groups. The high-resolution spectrum of N 1s could be deconvoluted into four smaller peaks with binding energies of 398, 399.6, 400.8 and 404.2 eV (Figure 2c), respectively, ascribed to N (C-N=C) groups (sp^2^ bonded), tertiary nitrogen N-(C)_3_ groups, C-N-H amino groups and charging effect [21]. Figure 2d displays the high-resolution spectrum of O 1s of the 65 O-CNS sample, which could be deconvoluted into two peaks centered at 531.5 and 532.5 eV, respectively, attributed to the bond of C-O and O-H [22,23]. In summary, the results mentioned above provide strong proof for the presence and chemical status of O ions in the 65 O-CNS sample. Additionally, vacancy defects could be correspondingly formed to compensate for the valence difference between N ions and O ions.

**Figure 3:**
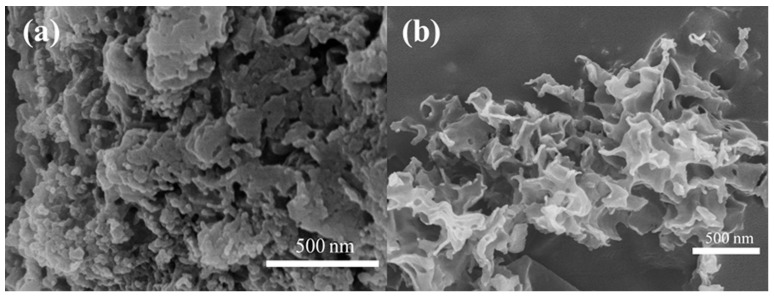
SEM images of (**a**) u-CNB and (**b**) 65 O-CNS.

Figure 3 shows the SEM images of uCNB and 65 OCNS samples. As shown in Figure 3a, the uCNB sample possessed a blocklike structure assembly with stacking layers. That is to say, the u-CNB sample obtained by direct calcination exhibited irregular block morphology, which was perhaps insufficient for providing abundant active sites for photocatalytic hydrogen production. Contrarily, a typical curled nanosheet-like structure appeared in the 65 OCNS sample, as seen in Figure 3b. Therefore, it is reasonably inferred that the addition of H_2_O_2_ mostly likely played a significant role in tuning the morphological structure. The features of such morphological structure would endow the 65 OCNS sample with a larger specific surface area, which is further beneficial for augmenting the number of active sites for the hydrogen production reaction.

**Figure 4:**
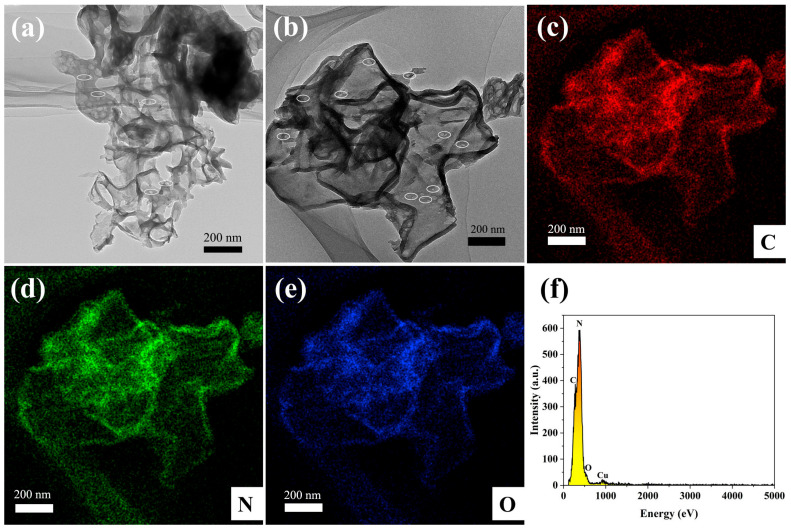
(**a**) TEM image, (**b**) HAADF-STEM image, (**c**–**e**) elemental mapping images of C, N and O elements, and (**f**) energy diffraction spectrum of 65 O-CNS.

The morphological structure of the 65 O-CNS photocatalyst was further examined by TEM and STEM images. As observed in Figure 4a, the 65 OCNS sample consisted of a mass of nanosheets with a relatively thin thickness of approximately 10 nm. Furthermore, it is clear from the magnified image (Figure 4b) that some irregular pores were distributed throughout thin nanosheets, resulting in the formation of a porous structure, which is beneficial for acquiring a large specific surface area. The formation of the porous structure was presumably associated with the bubbles produced during the polycondensation process with the assistance of H_2_O_2_. Therefore, one could come to the conclusion that the introduction of H_2_O_2_ indeed regulated the morphological structure. The element distribution of 65 O-CNS was revealed by high-angle annular dark-field (HAADF) and energy dispersive X-ray spectroscopy (EDS) element mapping, as illustrated in Figure 4c–f. It is clear that C, N and O elements (Figure 4c–f) were uniformly distributed throughout the 65 O-CNS sample, which further confirms the presence of the O element in the 65 O-CNS sample. As for the Cu element, it came from copper mesh in the sample preparation. Based on the above analysis, it can be concluded that the oxygen-doped C_3_N_4_ thin nanosheets with a porous structure were successfully prepared with H_2_O_2_ as the dopant source.

### 3.2. Specific Surface Area and Optical Properties

**Figure 5:**
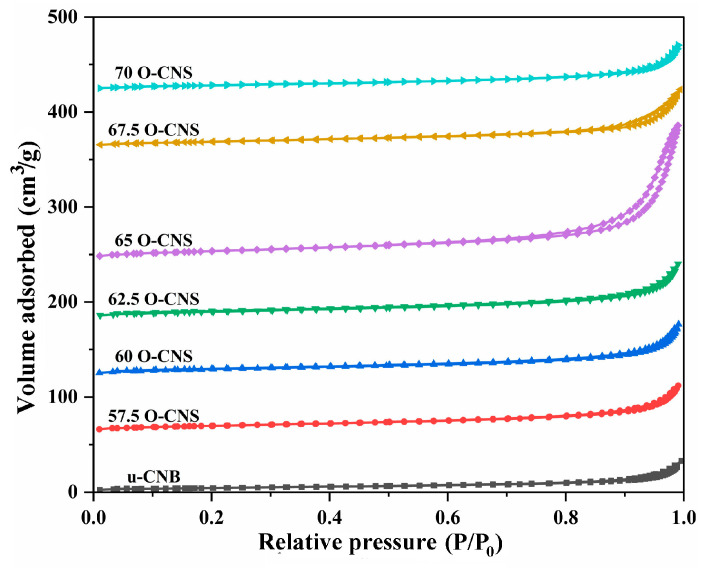
N_2_ adsorption–desorption isotherms of the as-obtained samples.

Figure 5 shows the N_2_ adsorption–desorption isotherms of u-CNB and different O-doped C_3_N_4_ samples. Obviously, the N_2_ adsorption–desorption isotherm for the O-doped C_3_N_4_ samples with a higher N_2_ volume adsorbed at high P/P_0_ were classified as type IV isotherms with H_3_ hysteresis loops, manifesting the mesoporous nature of the resulting sample. However, for the u-CNB sample, the lower volume of N_2_ adsorbed at high P/P_0_ was perhaps ascribed to the severe agglomeration of block-like particles [24]. Table 1 shows the specific surface area and pore size distribution of u-CNB and different O-doped C_3_N_4_ samples. Among them, u-CNB had the smallest specific surface area, attributable to the blocky structure formed by severe stacking of layers, while the 65 O-CNS sample possessed the largest specific surface area due to the nanosheets stacking. The increase in the specific surface area of the 65 O-CNS sample most likely increased the active sites for the catalytic reaction, which would facilitate the significant improvement in photocatalytic hydrogen production performance.

**Figure 6:**
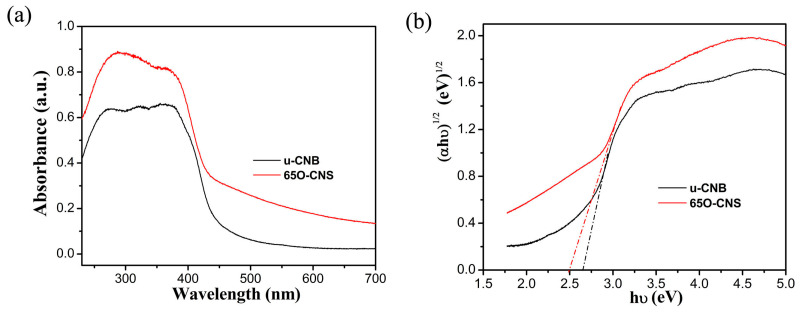
(**a**) UV–Vis DRS spectra and (**b**) Tauc plot of (αhv)^1/2^ vs. photon energy (hv) for bandgap energies of u-CNB and 65 O-CNS.

As is well known, light absorption capacity has a notable effect on photocatalytic hydrogen production performance. To compare the difference in the light absorption capacity between the uCBN and 65 OCNS samples, the UV–Vis diffuse reflection spectroscopy (DRS) spectra of the u-CNB and 65 O-CNS samples were obtained, and the results are shown in Figure 6a. Quite evidently, the intensity of light absorption for the 65 OCNS sample was significantly strengthened from the ultraviolet to the visible light region in comparison with that of u-CBN. In addition to the above, as compared with uCNB, the absorption edge of the 65 O-CNS sample was redshifted, indicating that the visible light absorption range was broadened due to the incorporation of the doping. By extrapolating the linear portion of the Tauc plots (Figure 6b), the bandgap energy (Eg) of u-CNB was determined to be approximately 2.62 eV, while the Eg of 65 O-CNS decreased to about 2.5 eV, indicating that the introduction of O atoms could regulate its electronic structure and enhance its light capture ability [25,26,27]. Thus, the improvement in the visible light absorption capacity was likely to increase the probability of producing more photogenerated carriers and further improve its catalytic performance.

### 3.3. Electron Paramagnetic Resonance and Electrochemical Measurements

**Figure 7:**
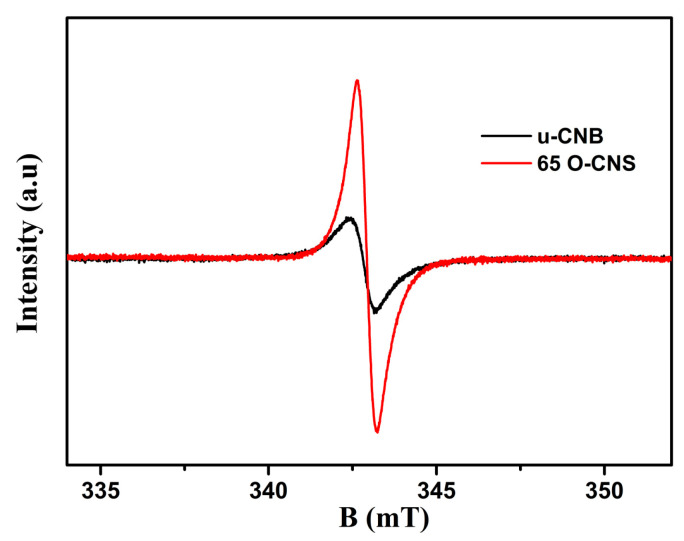
EPR spectra of u-CNB and 65 O-CNS.

The unpaired electrons of the prepared u-CNB and 65 O-CNS samples were tested by using electron paramagnetic resonance (EPR) at an ambient temperature and the result is shown in Figure 7. In the magnetic field from 333 to 353 mT, the u-CNB and 65 O-CNS samples had only one Lorentz curve centered around a g value of 2.003, which was considered a lone pair of electrons in sp^2^ hybrid carbon in g-C_3_N_4_ [28]. Moreover, the EPR signal intensity of the 65 OCNS sample was much higher than that of u-CNB, suggesting that more unpaired electrons in the 65 O-CNS sample would be generated under illumination. The result of EPR also revealed that the recombination of photogenerated electron–hole pairs for the 65 O-CNS sample was substantially inhibited, as compared with that of u-CBN.

**Figure 8:**
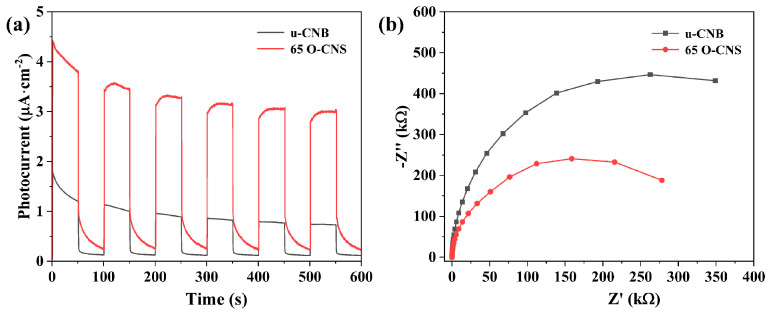
(**a**) Photocurrent curves and (**b**) electrochemical impedance spectra of u-CNB and 65 O-CNS electrodes.

As a previous research work has demonstrated [25], the photocurrent response of semiconducting photocatalysts is capable of reflecting the transfer and separation of photoexcited electrons and holes. The migration rate of photogenerated electron–hole pairs in the u-CNB and 65 O-CNS samples were further analyzed through electrochemical measurement. As presented in Figure 8a, a rapid and reversible photocurrent response sprouted for the u-CNB and 65 O-CNS samples, indicating their stability and reproducibility. The photocurrent density of the 65 O-CNS sample was much larger than that of u-CNB, signifying that the migration rate of photogenerated carriers of the 65 O-CNS sample was much higher than that of u-CNB. The above result also confirmed the remarkable elevation in the separation and migration rate of photoexcited carriers. Figure 8b presents the Nyquist plots of the electrochemical impedance spectra (EIS) for the u-CNB and 65 O-CNS samples. According to previous studies [29,30,31], the smaller the radius of the curve, the smaller the internal resistance. As expected, the impedance of the 65 O-CNS sample was smaller than that of u-CBN, revealing that more excited carriers could successfully migrate to the interface due to the restriction of carrier recombination. This result of the EIS was also in good accordance with the results of the photocurrent analysis. Based on the above, we can draw a conclusion that the migration rate of photogenerated carriers of the 65 O-CNS sample was remarkably accelerated and the recombination rate of photogenerated electron–hole pairs decreased significantly due to O doping.

### 3.4. Photocatalytic Hydrogen Production Performance

**Figure 9:**
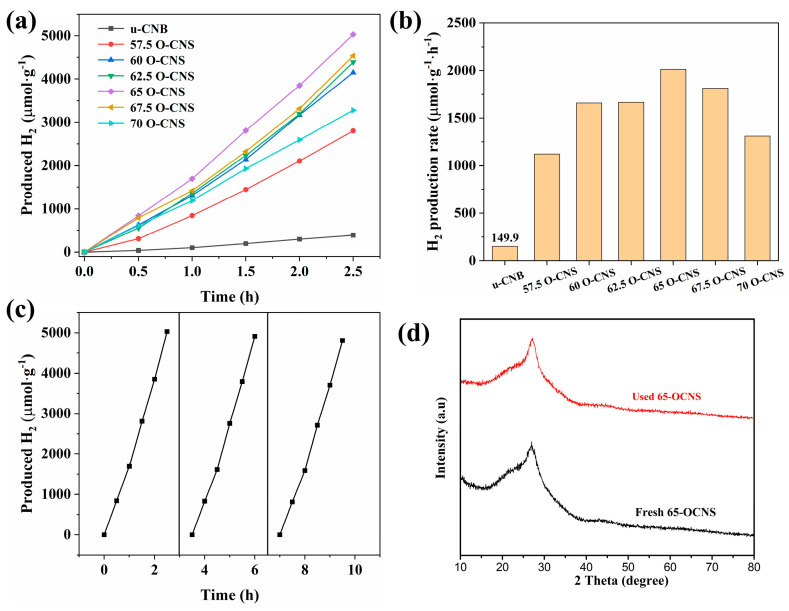
(**a**) Photocatalytic hydrogen production curves, (**b**) hydrogen production rate for u-CNB and O-CNS, (**c**) photocatalytic durability of 65 O-CNS and (**d**) XRD pattern of 65 O-CNS after cyclic runs.

The photocatalytic performance of different O-doped samples was evaluated by splitting water into hydrogen under visible light irradiation, and the results are shown in Figure 9. It can be seen from Figure 9a that the u-CNB sample had a very poor photocatalytic hydrogen production activity, with a hydrogen production rate of 149.9 μmol∙h^−1^∙g^−1^. Satisfactorily, the hydrogen production performance of the O-CNS samples was greatly improved with the increase in the H_2_O_2_ amount. However, when the quantity of H_2_O_2_ reaches 70 mL [32], the excess of H_2_O_2_ could result in the formation of more defects, which would act as recombination centers, leading to an increase in the recombination rate of photoinduced e^−^-h^+^ pairs. Accordingly, the hydrogen production rate of the O-doped CNS photocatalysts decreased when the loading amount of H_2_O_2_ exceeded the critical value of 65 mL. More importantly, the hydrogen production rate of the O-doped g-C_3_N_4_ was higher than that of most metal-free g-C_3_N_4_ photocatalysts under similar conditions (Table 2) [13,33,34,35,36,37]. In addition, cycling experiments with the 65 O-CNS sample were conducted. As observed in Figure 9c, the photocatalytic hydrogen production rate of the 65 O-CNS sample exhibited a slight decline after three cycle tests. Additionally, the XRD pattern in Figure 9d show that the crystal structure of 65 O-CNS exhibited no obvious change after cyclic experiments of photocatalytic hydrogen production. The above results indicate that the 65 O-CNS sample is a high-stability photocatalyst for photocatalytic hydrogen production.

### 3.5. Photocatalytic Mechanism

**Figure 10:**
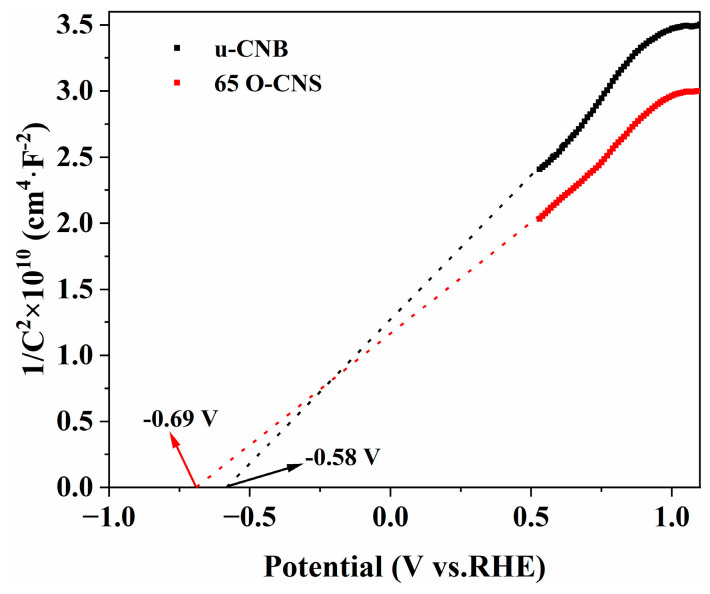
Mott–Schottky plots of u-CNB and 65 O-CNS.

In order to further investigate the enhancement mechanism of the photocatalytic activity of oxygen-doped C_3_N_4_, Mott–Schottky (M-S) plots of the u-CNB and 65 O-CNS samples were created and are depicted in Figure 10. The M-S plots of u-CNB and 65 O-CNS exhibit positive slopes, indicating the typical characteristics of n-type semiconductors. Based on the interception on the horizontal axis in the M-S plots, the flat potentials of u-CNB and 65 O-CNS were calculated to be −0.58 eV and −0.69 eV (vs. NHE), respectively. The 65 O-CNS sample showed a more negative flat band potential than u-CNB, indicating a larger amount of electron accumulation and a faster separation rate of photogenerated carriers in the 65 O-CNS photocatalyst. For n-type semiconductors, the potential of the flat band is about 0.2 eV more positive than that of the conduction band (CB) [38]. Therefore, the conduction band of u-CNB and 65 O-CNS was estimated to be −0.78 eV and −0.89 eV (vs. NHE), respectively. Combined with the UV–vis DRS results above, the valence band (VB) of the u-CNB and 65 O-CNS samples was calculated to be 1.84 eV and 1.61 eV (vs. NHE), respectively.

**Figure 11:**
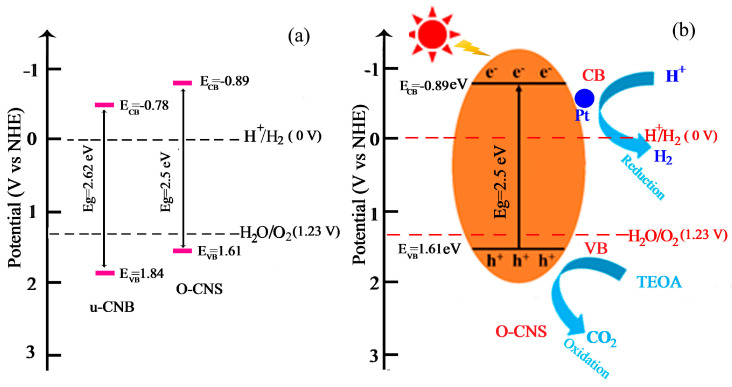
(**a**) Band structure of u-CNB and O-CNS; (**b**) enhancement mechanism of photocatalytic hydrogen production of O-CNS under visible light irradiation.

Based on the above calculation, the position of E_CB_ and E_VB_ can be determined and the schematic diagram for the band structure of u-CNB and 65O-CNS catalysts is presented in Figure 11a. It is obvious that the E_CB_ of O-CNS shifted upward from −0.78 V vs. NHE for U-CNB to −0.89 V vs. NHE for 65 O-CNS. Undoubtedly, a higher CB energy position would result in the promotion of the reduction capacity, and the decrease in Eg would lead to the enhancement of the visible light absorption capacity [39]. In light of the above demonstration, a proposed mechanism for the enhancement of photocatalytic behavior is schematically portrayed in Figure 11b. Upon exposure to visible light irradiation, the electrons (e^−^) in O-CNS are excited and migrate from the VB to CB, leaving holes in the VB (h^+^). Then, the excited electrons transfer from the CB of O-CNS to the surface of Pt particles. The accumulating electrons on the surface of Pt particles combine with hydrogen ions to produce H_2_. At the same time, TEOA is oxidized to form CO_2_ by photogenerated holes during the oxidation reaction process. The possible reaction equations involved in photocatalytic hydrogen evolution are as follows:

O-CNS + hv → h^+^ + e^−^ (1)

H_2_O + h^+^ → H^+^ + ·OH (2)

2e^−^ + 2H^+^ → H_2_ (3)

TEOA + h^+^ → CO_2_ (4)

TEOA + ·OH → CO_2_ (5)

Interestingly, with the doping of oxygen, the defect state could create active sites for the photoinduced charge’s excitation and induce bandgap narrowing, leading to the expansion of the photoresponsive range of O-CNS [40,41]. To summarize, the O doping induced the formation of a porous structure, the decrease in bandgap, the regulation of energy level, and the improvement in the separation and migration rate of photogenerated carriers. The porous structure could presumably achieve higher visible light absorption and scattering, provide more active sites, and speed up the release of hydrogen during the photocatalytic hydrogen process. The regulation of energy bands endowed O-CNS with a higher reduction capacity. The built energy levels formed by vacancy defects facilitated the efficient separation and transfer of photoexcited electrons. Therefore, the 65 O-CNS sample exhibited the highest photocatalytic hydrogen production rate due to the synergistic effect of the improved visible light absorption capacity, the augmented active sites, and the enhanced photogenerated carrier separation rate.

## 4. Conclusions

In the present work, oxygen-doped g-C_3_N_4_ porous nanosheets were achieved through an in situ, easily controllable and environmentally friendly approach, using H_2_O_2_ as an oxygen source. The modification of oxygen doping resulted in the formation of a porous structure and vacancy defects and boosted the visible light absorption capacity. The vacancy defects shaped into novel energy levels, which effectively restricted the recombination of photoinduced carriers. Simultaneously, the amount of O doping in g-C_3_N_4_ significantly affected the photocatalytic hydrogen evolution performance. Benefiting from the optimum amount of H_2_O_2_, the 65 O-CNS sample possessed the best photocatalytic hydrogen production performance. The notable improvement in the photocatalytic hydrogen evolution performance of the 65 O-CNS sample was mainly attributed to the porous structure, the increase in active sites, the boosted visible light absorption capacity and the enhancement of photogenerated charge carriers’ separation efficiency. This research work provides a new pathway for pursuing novel strategies for the preparation of element-doped C_3_N_4_ photocatalysts with exceptional photocatalytic hydrogen evolution performance.

## Figures and Tables

**Table 1:** The specific surface area, average pore width and pore volume of the samples.

Samples	BET (m^2^·g^−1^)	Average Pore Width (nm)	Pore Volume (cm^3^·g^−1^)
u-CNB	13.9	9.13	0.057
57.5 OCNS	33.8	27.26	0.33
60 OCNS	32.4	34.32	0.47
62.5 OCNS	34.5	40.54	0.54
65 OCNS	48.2	43.25	0.58
67.5 OCNS	31.3	46.78	0.61
70 OCNS	28.8	49.65	0.64

**Table 2:** Comparison of hydrogen evolution of element-doped g-C_3_N_4_ photocatalysts.

Samples	Light Source	Reactant Solution	Hydrogen Evolution (μmol h^−1^ g^−1^)	Reference
O-doped g-C_3_N_4_ nanosheets	300W Xe Lamp (λ > 420 nm)	80 mL water + 20 mL TEOA, 1% H_2_PtCl_6_	2012.9	This work
Pt/g-C_3_N_4_ nanotubes	300W Xe Lamp (λ > 420 nm)	TEOA aqueous solution (100 mL, 10 vol%)	5304	[33]
S-doped g-C_3_N_4_	300W Xe Lamp (λ > 420 nm)	50 mL aqueous TEOA solution (10 vol%), 2% Pt	161.32	[34]
Oxygen-doped g-C_3_N_4_ sheets	300 W Xenon lamp (λ > 420 nm)	100 mL aqueous solution containing 10 vol% TEOA, 3% Pt	2200	[15]
N-defective and S-doped g-C_3_N_4_	300 W Xe lamp (λ > 420 nm)	10 mL TEOA + 90 mL deionized water, 3% Pt	5651.5	[35]
Carbon-defective g-C_3_N_4_	300 W Xe lamp (λ > 420 nm)	100 mL of 10 vol% TEOA aqueous solution, 1% Pt	1534	[36]
K-doped g-C_3_N_4_	300 W Xe lamp (λ > 400 nm)	100 mL containing 10 vol % TEOA, 3% Pt	1337.2	[37]

## Data Availability

The data presented in this study are available in this article.
